# P2X_7_ Receptors as a Transducer in the Co-Occurrence of Neurological/Psychiatric and Cardiovascular Disorders: A Hypothesis

**DOI:** 10.1155/2009/545263

**Published:** 2009-08-10

**Authors:** Stephen D. Skaper, Pietro Giusti

**Affiliations:** Department of Pharmacology and Anesthesiology, University of Padova, Largo “E.Meneghetti” 2, 35131 Padova, Italy

## Abstract

*Background*. Over-stimulation of the purinergic P2X_7_ receptor may bring about cellular dysfunction and injury in settings of neurodegeneration, chronic inflammation, as well as in psychiatric and cardiovascular diseases. Here we speculate how P2X_7_ receptor over-activation may lead to the co-occurrence of neurological and psychiatric disorders with cardiovascular disorders. *Presentation*. We hypothesize that proinflammatory cytokines, in particular interleukin-1*β*, are key players in the pathophysiology of neurological, psychiatric, and cardiovascular diseases. Critically, this premise is based on a role for the P2X_7_ receptor in triggering a rise in these cytokines. Given the broad distribution of P2X_7_ receptors in nervous, immune, and vascular tissue cells, this receptor is proposed as central in linking the nervous, immune, and cardiovascular systems. *Testing*. Investigate, retrospectively, whether a bidirectional link can be established between illnesses with a proinflammatory component (e.g., inflammatory and chronic neuropathic pain) and cardiovascular disease, for example, hypertension, and whether patients treated with anti-inflammatory drugs have a lower incidence of disease complications. Positive outcome would indicate a prospective study to evaluate therapeutic efficacy of P2X_7_ receptor antagonists. *Implications*. It should be stressed that sufficient direct evidence does not exist at present supporting our hypothesis. However, a positive outcome would encourage the further development of P2X_7_ receptor antagonists and their application to limit the co-occurrence of neurological, psychiatric, and cardiovascular disorders.

## 1. Background

The P2X_7_ receptor (P2X_7_R) was originally described in cells of hematopoietic origin, and mediates the influx of Ca^2+^ and Na^+^ ions as well as the release of proinflammatory cytokines. P2X_7_Rs may affect cell death through their ability to regulate the processing and release of interleukin-1*β* (IL-1*β*), a key mediator in neurodegeneration, chronic inflammation, and, perhaps, some psychiatric diseases [1]. There is now ample evidence that elevated IL-1*β* levels, associated in many cases with P2X_7_R activation, occur in Alzheimer's disease, spinal cord injury, proinflammatory tissue trauma, neuropathic and inflammatory pain, and depressive illness. Preliminary, albeit intriguing observations suggest that elevated blood pressure may be associated with polymorphic variations in the *P2X_7_R* gene. Collectively, these findings have led us to propose a hypothesis in which the P2X_7_R is viewed as a common transducer of communication between the nervous, immune, and cardiovascular systems, whereby receptor over-activation may lead to the co-occurrence of neurological and psychiatric disorders with cardiovascular disorders, and vice versa.

## 2. Presentation of the Hypothesis

### 2.1. P2X_7_R as a Transducer in the Co-Occurrence of Neurological/Psychiatric and Cardiovascular Disorders

ATP-sensitive P2X_7_Rs are localized on cells of hematopoietic lineage including mast cells, erythrocytes, monocytes, peripheral macrophages, dendritic cells, T- and B-lymphocytes, epidermal Langerhans cells, and glial cells in the CNS [2, 3]. Activation of P2X_7_Rs leads to rapid changes in intracellular calcium concentrations, release of the proinflammatory cytokine IL-1*β* and following prolonged exposure, the formation of cytotoxic pores in plasma membranes. P2X_7_Rs could affect IL-1*β* also via the 5-lipoxygenase pathway; that is, P2X_7_R activation leads to leukotriene formation (e.g., in astrocytes) [4] and leukotrienes increase IL-1*β* expression and release [5]. Both the localization and functional consequences of P2X_7_R activation indicate a role in inflammatory processes. Activated immune cells (lymphocytes) [6], macrophages [7], microglia [8], and platelets [9], and dying cells may release high concentrations of ATP into the extracellular space [10], while extracellular ATP concentrations increase under inflammatory conditions in vivo [11] and in response to tissue trauma [12]. In addition, pro-inflammatory cytokines and bacterial products upregulate P2X_7_R expression and increase its sensitivity to extracellular ATP [13]. 

**Figure 1:**
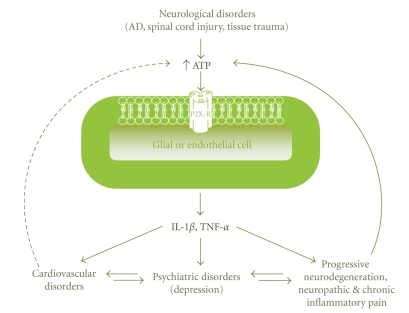
Schematic representation of potential interactions between the cardiovascular and nervous systems, which may lead to the co-occurrence of cardiovascular, neurological, and psychiatric disorders. In this hypothesis, the P2X_7_ purinergic receptor plays a pivotal role in linking these disorders, as a result of elevated levels of extracellular ATP and the release of pro-inflammatory cytokines such as interleukin-1*β* (IL-1*β*) and tumor necrosis factor-*α* (TNF-*α*). AD, Alzheimer's disease.

We hypothesize that pro-inflammatory cytokines, in particular IL-1*β*, are key players in the pathophysiology of neurological, psychiatric, and cardiovascular diseases. Critically, this premise is based on a role for the P2X_7_R in triggering a rise in these cytokines. One of the most striking features of ATP is its unmatched ability to promote massive release of mature IL-1*β* from lipopolysaccaride primed mononuclear phagocytes and other cell types, including microglia [14]. ATP-driven maturation and release of IL-1*β* are specifically mediated by the P2X_7_ receptor for extracellular ATP [15, 16]. Given the broad distribution of P2X_7_Rs in nervous, immune, and vascular tissue cells, this receptor is proposed as playing a common transductional role in linking the nervous, immune, and cardiovascular systems. We also hypothesize that P2X_7_R over-activation may lead to the co-occurrence of neurological and psychiatric disorders with cardiovascular disorders (Figure 1). 

These speculative hypotheses are based on an extensive body of published studies describing pro-inflammatory cytokine elevations and P2X_7_R over-activity in neurodegenerative diseases, pain, depression, and cardiovascular disease. Activation of P2X_7_Rs provides an inflammatory stimulus [17], and P2X_7_R-deficient mice have substantially attenuated inflammatory responses [15, 18]. Acute spinal cord injuries produce highly inflammatory environments [19]. In rats subjected to spinal cord injury, areas surrounding the traumatic lesion displayed an abnormally high and sustained pattern of ATP release, and delivery of a P2X_7_R antagonist after acute impact injury improved functional recovery and diminished cell death in the peritraumatic zone [20]. P2X_7_R-like immunoreactivity was upregulated around *β*-amyloid plaques in a transgenic mouse model of Alzheimer's disease, and was regionally localized with activated microglia and astrocytes [21]. Up-regulation of P2X_7_Rs on microglia is seen after ischemia in the cerebral cortex of rats [22], and on reactive astrocytes in multiple sclerosis autopsy brain tissue [23]. Genetic and pharmacological approaches have been used to show that P2X_7_R activation on microglia is necessary for microglial cell-mediated injury of neurons [24]. 

 Phenotypic data from P2X_7_R null mice provide important evidence for participation of this channel in proinflammatory tissue trauma. There is a lower incidence and severity of collagen antibody-induced arthritis in P2X_7_R knockout mice [25], and inflammatory and neuropathic hypersensitivity is completely absent to both mechanical and thermal stimuli in these mice [18]. Moreover, P2X_7_R is upregulated in human dorsal root ganglia and injured nerves obtained from chronic neuropathic pain patients [18]. Endogenous IL-1 levels are increased in the nervous system in response to trauma associated with mechanical damage, ischemia, seizures, and hyperexcitability [26]. 

 There appears to be a strong relationship between depression and immunological dysfunction in depressed patients [27]. Cytokines like IL-1*β* are suggested to be involved in the pathophysiology of depression, and excessive secretion of macrophage cytokines (IL-1*β*, tumor necrosis factor-*α*, interferon-*γ* could be a potential causative factor [28]. Central and systemic administration of proinflammatory cytokines to animals induces “sickness behavior”, which is characterized by many of the physiological and behavioral changes associated with depression [27, 29]. Clinical use of cytokines (e.g., interferon-*α*) produces depressive-like symptoms that can be attenuated with antidepressant treatment [30], and major depressive illness is associated with significant elevations in the density of microglia and hypersecretion of proinflammatory cytokines, suggesting that the latter could be involved in the etiopathogenesis of depression [31–34]. 

 Apoptotic cell death occurs in a number of vascular diseases, including atherosclerosis and hypertension [35]. Shear stress that occurs during changes in blood flow causes a substantial release of ATP from vascular endothelial cells [36]. ATP may also be released from cardiomyocytes in ischemic or hypoxic conditions [37]. P2X_7_R-associated production of proinflammatory cytokines like tumor necrosis factor-*α* could promote endothelial cell apoptosis [34], and play a role in vascular remodeling in hypertension [38]. P2X receptor channels are involved in transducing aldosterone-mediated signaling in the distal renal tubule and are potential candidate genes for blood pressure regulation [39]. On an intriguing note, there is evidence to suggest that elevated nighttime diastolic blood pressure is associated with single nucleotide polymorphisms of the *P2X_7_R* gene [40]. P2X_7_Rs are expressed in human saphenous vein myocytes [41], and venous diseases may favor conditions allowing P2X_7_R activation and lysis of venous myocytes. ATP released after hypoxia, stress and inflammation, or membrane damage, conditions found in the vessel wall of varicose veins, may lead to P2X_7_R-induced pore formation, the disorganization and loss of contractile myocytes in the muscle layers of the media of varicose veins, and venous disease. 

 Fibroblasts are a key structural element of the arterial wall known to play a major role in atherosclerosis and diabetic angiopathy [42]. Fibroblasts from type-2 diabetes patients are characterized by a hyperactive purinergic loop [43].

## 3. Testing the Hypothesis

Retrospective studies inform us, for example, that depression is recognized as having high prevalence in several medical conditions including infectious, autoimmune, and neurodegenerative diseases, conditions associated with a proinflammatory status [28, 44]. Increasing evidence now points to a strong relationship between depression and immunological dysfunction in depressed patients, while clinical use of cytokines produces depressive-like symptoms responsive to antidepressant treatment [30]. While depression and cardiovascular comorbidity have been recognized for some time [45], a proinflammatory link has only recently been investigated [46]. Although a first step, these correlations are not definitive proof of our concept. More extensive prospective studies are required to confirm the above, and to investigate whether a link exists between illnesses with a proinflammatory component (e.g., inflammatory and chronic neuropathic pain) and cardiovascular disease, for example, hypertension, and whether patients treated with anti-inflammatory drugs have a lower incidence of cardiovascular complications. This would then need to be followed with a demonstration that pharmacological block of P2X_7_Rs provides therapeutic benefit in these conditions.

## 4. Implications of the Hypothesis

If a strong link between neurological, psychiatric and, cardiovascular disorders could be established, then within this framework P2X_7_R activity can be viewed as playing a common transductional (“gatekeeper”) role in the development of comorbidity between the nervous, immune, and cardiovascular systems. The outcome, if positive, would provide the impetus for further development and clinical application of selective and potent P2X_7_R antagonists.
